# Temporal trends in serum concentrations of polychlorinated dioxins, furans, and PCBs among adult women living in Chapaevsk, Russia: a longitudinal study from 2000 to 2009

**DOI:** 10.1186/1476-069X-10-62

**Published:** 2011-06-22

**Authors:** Olivier Humblet, Oleg Sergeyev, Larisa Altshul, Susan A Korrick, Paige L Williams, Claude Emond, Linda S Birnbaum, Jane S Burns, Mary M Lee, Boris Revich, Andrey Shelepchikov, Denis Feshin, Russ Hauser

**Affiliations:** 1 Environmental and Occupational Medicine and Epidemiology Program, Department of Environmental Health, Harvard School of Public Health, Boston, Massachusetts, USA; 2 Department of Physical Education and Health, Samara State Medical University, Samara, Russia; 3 Chapaevsk Medical Association, Chapaevsk, Russia; 4 Department of Environmental Health, Harvard School of Public Health, Boston, Massachusetts; 5 Environmental Health and Engineering, Inc., Needham, Massachusetts, USA; 6 Channing Laboratory, Department of Medicine, Brigham and Women's Hospital and Harvard Medical School, Boston, Massachusetts, USA; 7 Department of Biostatistics, Harvard School of Public Health, Boston, Massachusetts, USA; 8 Department of Environmental and Occupational Health, Faculty of Medicine, University of Montreal, Montreal, Quebec, Canada; 9 BioSimulation Consulting Inc., Newark, Delaware, USA; 10 National Cancer Institute, National Institutes of Health, Department of Health and Human Services, Bethesda, Maryland, USA; 11 Pediatric Endocrinology Division, Departments of Pediatrics and Cell Biology, University of Massachusetts Medical School, Worcester, Massachusetts, USA; 12 Center for Demography and Human Ecology, Institute for Forecasting, Russian Academy of Sciences, Moscow, Russia; 13 Institute of Ecology and Evolution of Russian Academy of Sciences, Moscow, Russia

## Abstract

**Background:**

The present study assessed the temporal trend in serum concentrations of polychlorinated dibenzo-*p*-dioxins, dibenzofurans, and biphenyls (PCBs) among residents of a Russian town where levels of these chemicals are elevated due to prior industrial activity.

**Methods:**

Two serum samples were collected from eight adult women (in 2000 and 2009), and analyzed with gas chromatography-high-resolution mass spectrometry.

**Results:**

The average total toxic equivalency (TEQ) decreased by 30% (from 36 to 25 pg/g lipid), and the average sum of PCB congeners decreased by 19% (from 291 to 211 ng/g lipid). Total TEQs decreased for seven of the eight women, and the sum of PCBs decreased for six of eight women. During this nine year period, larger decreases in serum TEQs and PCBs were found in women with greater increases in body mass index.

**Conclusions:**

This study provides suggestive evidence that average serum concentrations of dioxins, furans, and PCBs are decreasing over time among residents of this town.

## Introduction

Polychlorinated dibenzo-*p*-dioxins (PCDDs), dibenzofurans (PCDFs), and biphenyls (PCBs) are classes of toxic organochlorine compounds which are ubiquitous in the environment [1,2]. They are lipophilic and persistent, and their elimination from the body can take years or even decades [3]. Current human exposure is mostly through diet [4], although some populations may have continuing environmental exposures due to past or current industrial activity [5-8].

Numerous studies in the general population have reported that serum concentrations of these chemicals correlate positively with age [9,10], but also that average population levels are declining over time [11]. The objective of this study was to characterize temporal trends in serum concentrations of PCDD/PCDF/PCBs among reproductive age women living in Chapaevsk, Russia. Elevated levels of these compounds have been detected in serum samples from Chapaevsk residents [12,13], as well as in environmental samples and food [14]. For over 50 years this town was the site of chlorinated chemical production at the Middle Volga Chemical Plant [7], also known as SVZH. Plant activity started decreasing in 1991 [15], and ceased in 2003. The plant produced chemical blister agents (mustard gas and lewisite) prior to 1949, hexachlorocyclohexane (lindane) and its derivatives from 1967 to 1987, then crop protection chemicals (liquid chlorine, acids, methyl chloroform, and vinyl chloride) until its closure [7]. Dioxins were generated as unwanted by-products of chemical manufacturing [7], whereas PCBs were typically used in electrical capacitors and transformers, although their specific use at SVZH is unknown. Environmental release of these chemicals most likely occurred during incineration and disposal of hazardous waste from the plant. We collected serum samples nine years apart (in 2000 and 2009) from eight non-occupationally exposed women living in Chapaevsk, and in 2009 all samples were sent for analysis of PCDD/PCDF/PCBs.

## Methods

### Study population

Ten healthy women were recruited in 2000 for a pilot feasibility study on early pregnancy loss, at which time a serum sample was collected and archived, and a health and lifestyle questionnaire was completed. Newly married women in Chapaevsk were invited to join, and 10 enrolled in the pilot project. To be included in the original study women needed to be married, age 20-34 years, and trying to conceive. To investigate temporal trends in PCDD/PCDF/PCB exposure, participants were re-contacted in 2009, and a second serum sample and lifestyle questionnaire was collected from eight of the ten women (of the two others, one had left Chapaevsk, and one declined to participate).

The research protocol was approved by the Human Studies Institutional Review Boards of the Chapaevsk Medical Association, UMass Medical School, and Harvard School of Public Health. All participants gave their consent before participating in each of the two assessments.

### Measurement of dioxin, furan, and PCB serum concentrations

Samples were stored at -20 degrees C until analysis. All 16 serum samples (10-15 ml each) were analyzed in 2009 using isotope dilution gas chromatography-high-resolution mass spectrometry (GC-HRMS-ID) at the Laboratory of Analytical Toxicology of the Severtsov Institute of Ecology and Evolution in Moscow, Russia. The list of dioxin-like congeners (called dioxins in this manuscript) included 7 PCDDs, 10 PCDFs, 4 co-planar PCBs, and 6 mono-ortho PCBs. The list of non-dioxin like PCBs (NDL-PCBs) included congeners 18, 28, 44, 49, 52, 66, 74, 87, 99, 101, 110, 128, 138, 146, 149, 151, 153, 170, 172, 177, 178, 180, 183, 187, 190, 194, 195, 196, 199, 203, 206, 209. Congeners are identified according to the International Union for Pure and Applied Chemistry (IUPAC) nomenclature.

Prior to analysis of study samples the laboratory carefully cleaned and checked all reagents, solvents, and glassware, and analyzed a method blank to confirm that it was free from interference. Then each set of four samples was extracted and analyzed with one method blank, which was treated identically to the serum samples.

For the analysis of dioxins, furans and co-planar PCBs the serum samples were spiked with a mixture of ^13^C_12_-labeled PCDDs/PCDFs and PCBs as internal standards. Methods for sample preparation, clean up and analysis follow US Environmental Protection Agency (EPA) methods 1613 [16] and 1668 [17]. Analysis was performed using a Thermo Finnigan MAT 95XP. The analytes were separated on a capillary column (SGE-BPX5, 30 meters, 0.25 mm, 0.25 microns) and quantified using selected-ion-monitoring high resolution (10,000 resolving power) mass spectrometry (HRGC-ID/HRMS). Quantification was by isotope dilution mass spectrometry using calibration standards containing ^13^C labeled and unlabeled analytes. Mono-*ortho *(M-PCBs) and non-dioxin-like PCBs were separated on a capillary column (SGE-HT8, 30 meters, 0.25 mm, 0.25 microns).

LODs were calculated as three times chromatographic noise. The LODs for dioxins and furans ranged from 0.01 to 0.04 pg/g, and for C-PCBs from 0.1 to 0.6 pg/g serum. The levels of target analytes in 4 blanks analyzed with study samples were below the LODs. The mean (SD) recoveries were 90% (5.5) for TCDD and 84% (3.1) for HxCDD.

Serum total cholesterol and triglycerides were measured with an enzymatic colorimetric assay using the COBAS INTEGRA 400 plus system, and the serum total lipid content was calculated using the Phillips equation [18]. All PCDD/PCDF/PCB serum concentrations were lipid-adjusted. Individual congener concentrations below the limit of detection (LOD) were assigned a value equal to the LOD divided by the square root of 2 [19,20].

The serum concentrations of PCDD/PCDF/PCBs were summarized with two measures: the total sum of toxic equivalencies (i.e. total TEQ [21]) for PCDDs, PCDFs, co-planar PCBs (C-PCBs) and mono-*ortho *PCBs (M-PCBs); and the sum of all 36 assayed non-coplanar PCB concentrations (i.e. ∑PCBs).

### Statistical methods

Pearson correlation coefficients were calculated for the log10-transformed serum concentrations in 2000 and 2009; 95% confidence intervals (CIs) for the correlation coefficients were calculated using the method of Shen and Lu [22].

## Results

### Demographics

The women were aged 21 to 27 years at initial enrollment, and 30 to 35 years at the second follow-up in 2009. All had lived exclusively in Chapaevsk since 2000. One woman moved to Chapaevsk in 2000; the others had spent a maximum of 2 years living outside of Chapaevsk. None were employed at SVZH, the local chemical plant. BMIs ranged from 17 to 30 at enrollment in 2000, and from 20 to 31 in 2009. Seven of the eight women had a body mass index (BMI) <25 kg/m^2 ^in 2000; five did so in 2009. Six of eight women breastfed (either 1 or 2 children) between 2000 and 2009, with total durations ranging from 3 to 30 months (Table 1). One woman was currently breastfeeding; all others had last breastfed in 2007 or earlier. The two women who did not breastfeed were nulliparous.

**Table 1:** Individual characteristics and serum concentrations of total TEQs and ∑PCBs, in 2000 and 2009, among 8 Russian women

				Total TEQs (pg/g lipid)			∑ PCBs (ng/g lipid)		
				
	BMI percent change, 2000 to 2009	BMI in 2009	Months of breastfeeding, 2000 to 2009	2000	2009	Percent change, 2000 to 2009	2000	2009	Percent change, 2000 to 2009
1	-3.0%	19.7	>12	16.7	14.7	-12%	139	215	+54%

2	0.4%	22.7	>12	39.5	19.7	-50%	327	185	-43%

3	3.7%	31.2	<6	29.2	31.2	+7%	238	290	+22%

4	8.4%	24.5	0	89.7	60.9	-32%	210	173	-18%

5	11.3%	19.7	>12	27.5	19.6	-29%	408	232	-43%

6	13.9%	27.0	6-12	28.3	19.9	-30%	388	266	-31%

7	22.9%	21.5	0	25.1	15.5	-38%	336	175	-48%

8	28.6%	25.6	>12	32.6	13.6	-58%	280	149	-47%

*Mean (SD):*	11% (11)	24 (4)	11 (10)	36 (23)	25 (16)	-30% (21)	291 (92)	211 (49)	-19% (38)

All percent changes are calculated using the 2000 values as the denominator.

### Serum concentrations of total TEQs and ∑PCBs

The total TEQ and ∑PCB serum concentrations in both 2000 and 2009 are shown in Table 1 (results for all measured congeners are shown in Additional File 1, Table S1). The mean serum concentrations in 2000 and 2009 were, for total TEQs, 36 and 25 pg/g lipid, and for ∑PCBs, 291 and 211 ng/g lipid, respectively. Excluding the one woman who moved to Chapaevsk in 2000 (and therefore does not reflect the exposure levels of long-term residents), the respective 2000 and 2009 mean serum concentrations were, for total TEQs, 39 and 26 pg/g lipid, and for ∑PCBs, 312 and 210 ng/g lipid.

**Figure 1:**
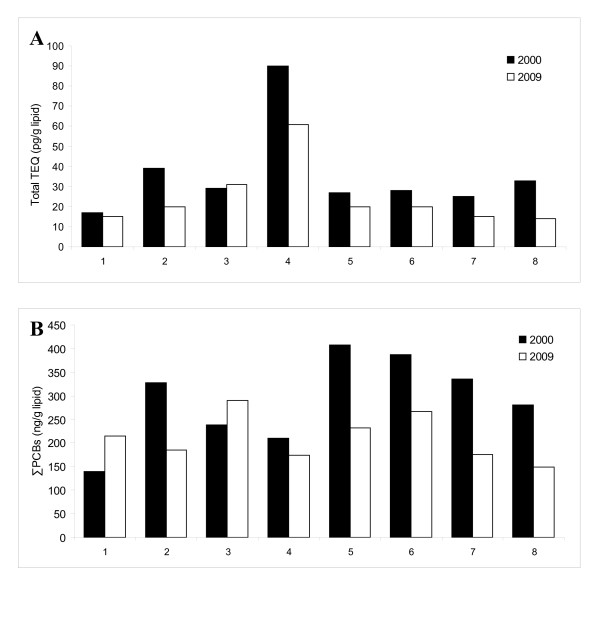
**Serum concentrations of serum total TEQs and ∑ PCBs among eight Russian women, in 2000 and 2009**. Figure 1A shows the serum total TEQ concentrations (pg/g lipid). Figure 1B shows the serum ∑PCB concentrations (ng/g lipid). Participants are numbered as in Table 1.

Trends in total TEQs and PCBs between 2000 and 2009 for each individual are shown graphically in Figure 1. Decreasing concentrations were found for seven of the eight women for TEQs, and for six of the eight women for ∑PCBs. The mean percent change was -30% for total TEQs, and -19% for ∑PCBs (Table 1). The mean decreases in the TEQs of the subtypes of dioxin-like compounds were -42% for PCDDs, -25% for PCDFs, -24% for M-PCBs, and -14% for C-PCBs. Similar mean decreases were seen when concentrations were expressed as the sum of measured congener values instead of in TEQs: -37% for PCDDs, -35% for PCDFs, -24% for M-PCBs, and -1% for C-PCBs. The slightly smaller decrease in C-PCB concentrations than for C-PCB TEQs (i.e. -1% vs. -14%) is because concentrations of PCB77 increased for 4 of the 8 women, but the small TEF of this congener (i.e. 0.0001) resulted in the increase having little effect on the mean C-PCB TEQs.

Several individual characteristics were associated with the magnitude of the changes in serum concentration between 2000 and 2009 (although the small sample size precluded formal statistical analyses on these predictors). First, a greater decrease in both total TEQs and ∑PCBs was observed among the four women with the largest increases in BMI during this nine year period, as compared to the other four women. TEQs decreased by an average of 39% among women whose BMIs increased by 10% or more, compared to 22% among women whose BMIs increased <10% or decreased. Similarly, PCBs decreased by an average of 42% among the former group of women, but increased by 3.8% among the latter. The latter group also includes the only woman's whose BMI was >30 kg/m^2 ^at either assessment. Second, the four women who breastfed longest (i.e. ≥12 months) had larger average decreases in TEQs than the women who breastfed less (-37% vs. -23% for TEQs, respectively), but there was not a greater decrease in PCBs by breastfeeding duration (-20% vs -19%, respectively).

### Correlations between serum concentrations in 2000 and 2009

The Pearson correlations between the log10-transformed serum concentrations in 2000 and 2009 were 0.3 (95% CI: -0.5; 0.8) for total TEQs (after excluding the highest value, which had inflated the correlation to 0.8), and 0.1 (95% CI: -0.6; 0.7) for ∑PCBs. However, the interpretability of these correlations was limited by the extremely wide confidence intervals.

## Discussion

### Summary

This study consisted of eight women from a town in Russia with high exposure to dioxins and PCBs from non-occupational sources. Each woman's serum concentration of dioxins and PCBs was assessed in both 2000 and 2009. All of the women's serum concentrations of both total TEQs and ∑PCBs were greater than the 90^th ^percentile values for US women 20 to 39 years of age in 2003-2004 (i.e. 16.2 pg/g lipid for total TEQs, and 157.8 ng/g lipid for ∑PCBs [10]) For almost all of the women, serum concentrations of total TEQs and ∑PCBs decreased substantially between 2000 and 2009 and, as expected, larger decreases were seen among women with greater BMI increases [23].

Although one must exercise caution in making inferences to the entire population of Chapaevsk (pop. 72,000) from the decreases observed among these eight women, concordant results were seen in our recent cross-sectional study among Chapaevsk women [13]. 446 adult women's serum concentrations of PCDD/PCDF/PCBs were collected between 2003 and 2005, with average serum concentrations similar to those seen here. Levels were lower among women sampled later in the study period, adjusting for all other predictors. In multivariate linear regressions a one-year later blood draw was associated with 9.8% lower mean serum concentrations of ∑PCBs, and 10.5% lower mean total TEQs. While these age-adjusted regression results should not be interpreted as predictive of longitudinal changes in serum concentrations, they do suggest a trend similar to what was observed among the eight women.

### Strengths and limitations

This study is one of the few to measure repeated serum concentrations of PCDD/PCDF/PCBs in a non-occupationally and non-accidentally exposed cohort. A particular strength of this study is that serum samples from both 2000 and 2009 were analyzed at the same time, precluding any potential bias from laboratory drift. Dioxins and PCBs are highly stable in stored serum [24], thus little degradation would be expected in the samples stored since 2000. However, the small sample size of eight women warrants that these findings be interpreted with caution. Furthermore, no longitudinal data on consumption of local meat or produce was available to assess the contribution of changing dietary patterns to the temporal changes in TEQ and PCB serum concentrations observed here.

## Conclusions

This study reports the serum dioxin and PCB concentrations of adult women from a Russian town with high levels of non-occupational exposure to these compounds. All the women's levels were higher than typically seen in the US. Each woman was sampled twice: in 2000 and again in 2009. Among most of the women, serum concentrations of dioxins and PCBs decreased over this time period. Decreases were seen for all classes of dioxin-like compounds (i.e. PCDDs, PCDFs, C-PCBs and M-PCBs).

These results, while based on a small number of women, are consistent with those from our large cross-sectional study recently conducted among women in the same town. In the latter study, serum concentrations of dioxins and PCBs were lower, on average, among women sampled later in the study period, adjusting for all other predictors. While it is known that a general trend towards decreasing dioxin and PCB levels exists in the general population [25,26], it is significant that this also appears to be true among women living in an environment with a substantial, and relatively recent (SVZH ceased operations in 2003, although production started decreasing in 1991), non-occupational exposure source.

## List of abbreviations

BMI: Body mass index kg/m^2^; C: Celsius; CI: Confidence interval; C-PCBs: Co-planar PCBs; LOD: limit of detection; M-PCBs: Mono-*ortho *PCBs; ng/g: nanogram per gram, 10^-9 ^g; PCBs: Polychlorinated biphenyls; PCDDs: Polychlorinated dibenzo-*p*-dioxins; PCDFs: Polychlorinated dibenzofurans; pg/g: picogram per gram, 10^-12 ^g; SD: Standard deviation; SVZH: Middle Volga Chemical Plant; TEQ: Toxic equivalents; WHO: World Health Organization;

## Competing interests

The authors declare that they have no competing interests.

## Authors' contributions

OH participated in the study design, conducted the data analysis, and drafted the manuscript. OS supervised study recruitment and collection of blood samples and questionnaire data, and contributed to the drafting of the manuscript and the revisions. LA participated in the study design and coordination, and contributed to the manuscript revisions. SK, PW, CE, LB, JB, and ML assisted with the interpretation of the data and contributed to the drafting of the manuscript and the revisions. BR facilitated the data collection and reviewed the manuscript. AS and DF conducted the mass spectroscopy analyses and contributed to the manuscript revisions. RH conceived of the study, participated in its design and coordination, and helped draft and revise the manuscript. All authors read and approved the final version.

## Supplementary Material

Additional file 1 **Table s1**. The supplementary data table shows the distributions of all measured serum concentrations of polychlorinated dibenzo-*p*-dioxin, polychlorinated dibenzofuran, and polychlorinated biphenyl congeners, in 2000 and 2009, among the 8 study participants. Click here for file
